# Construction and validation of an early warning model for predicting the acute kidney injury in elderly patients with sepsis

**DOI:** 10.1007/s40520-022-02236-3

**Published:** 2022-09-02

**Authors:** Qi Xin, Tonghui Xie, Rui Chen, Hai Wang, Xing Zhang, Shufeng Wang, Chang Liu, Jingyao Zhang

**Affiliations:** 1 Department of Hepatobiliary Surgery, The First Affiliated Hospital of Xi’an Jiaotong University, Xi’an, 710061 Shaanxi China; 2 Department of SICU, The First Affiliated Hospital of Xi’an Jiaotong University, Xi’an, 710061 Shaanxi China; 3 Department of General Surgery, The First Affiliated Hospital of Xi’an Jiaotong University, 277 Yanta West Road, Xi’an, 710061 China

**Keywords:** Acute kidney injury, Elderly sepsis patients, Early warning, Nomogram

## Abstract

**Background:**

Sepsis-induced acute kidney injury (S-AKI) is a significant complication and is associated with an increased risk of mortality, especially in elderly patients with sepsis. However, there are no reliable and robust predictive models to identify high-risk patients likely to develop S-AKI. We aimed to develop a nomogram to predict S-AKI in elderly sepsis patients and help physicians make personalized management within 24 h of admission.

**Methods:**

A total of 849 elderly sepsis patients from the First Affiliated Hospital of Xi’an Jiaotong University were identified and randomly divided into a training set (75%, *n* = 637) and a validation set (25%, *n* = 212). Univariate and multivariate logistic regression analyses were performed to identify the independent predictors of S-AKI. The corresponding nomogram was constructed based on those predictors. The calibration curve, receiver operating characteristics (ROC)curve, and decision curve analysis were performed to evaluate the nomogram. The secondary outcome was 30-day mortality and major adverse kidney events within 30 days (MAKE30). MAKE30 were a composite of death, new renal replacement therapy (RRT), or persistent renal dysfunction (PRD).

**Results:**

The independent predictors for nomogram construction were mean arterial pressure (MAP), serum procalcitonin (PCT), and platelet (PLT), prothrombin time activity (PTA), albumin globulin ratio (AGR), and creatinine (Cr). The predictive model had satisfactory discrimination with an area under the curve (AUC) of 0.852–0.858 in the training and validation cohorts, respectively. The nomogram showed good calibration and clinical application according to the calibration curve and decision curve analysis. Furthermore, the prediction model had perfect predictive power for predicting 30-day mortality (AUC = 0.813) and MAKE30 (AUC = 0.823) in elderly sepsis patients.

**Conclusion:**

The proposed nomogram can quickly and effectively predict S-AKI risk in elderly sepsis patients within 24 h after admission, providing information for clinicians to make personalized interventions.

**Supplementary Information:**

The online version contains supplementary material available at 10.1007/s40520-022-02236-3.

## Introduction

From 2000 to 2019, the average global life span grew by more than 6 years, with life expectancy growing from 66.8 to 73.4 years [1]. As the world's population ages, so does the demand for admission to hospitals and to intensive care units (ICU). In Australia, for example, the total number of hospital admissions for the oldest patients (85 years) almost doubled from 2005 to 2015 [1]. Moreover, in Northern Denmark, 74% and 12.6% of the patients admitted to the ICU are older than 50 years and 80 years, respectively [2].

Sepsis is defined as lethal organ dysfunction caused by a dysregulated host response to infection [3]. The incidence of sepsis is dramatically increased (58–65%) in elderly patients, and age is an independent predictor of sepsis mortality [4]. Some studies showed that sepsis mortality increased from 10% in children to 26% in patients aged 60–64 and 38% in those over 85 [4]. In addition, the elderly are the primary age group affected by AKI [5]. The kidney is also a particularly vulnerable organ to sepsis, and S-AKI is associated with a high mortality rate in sepsis patients [6]. The primary processes of AKI are decreased renal blood flow, subsequent tubular epithelial cell death, or acute tubular necrosis. Although the detailed mechanisms underlying the pathogenesis of S-AKI in elderly sepsis patients remain unclear, early detection of S-AKI and timely treatment may improve the clinical prognosis.

Many researchers have focused on the independent predictors of S-AKI in elderly sepsis patients over the past decades. For example, the delta neutrophil index (DNI), proenkephalin (PENK), urinary interleukin-18, urinary KIM-1, and neutrophil gelatinase-associated lipocalin (NGAL) have been proposed to diagnose AKI [7–9]. Unfortunately, these novel biomarkers are not only rarely available but often require a high cost. Moreover, several risk factors for developing AKI in sepsis patients have been studied, including older age, platelet counts, white blood cell (WBC), PTA, and PCT levels [10–12]. However, these markers often lack sensitivity or specificity when used as a single index. Currently, there are no reliable and robust predicted models to identify S-AKI in elderly sepsis patients by combining these factors. Additionally, several studies have used MAKE30 as a test endpoint for conducting clinical trials, such as the SALT, SMART, and pediatric sepsis trials [13]. The predictive models also could be used to stratify patients and determine the feasibility of 30-day mortality and MAKE30 as endpoints in future clinical trials. Thus, it is of great clinical value to construct a nomogram by using routine information, which can provide an evidence-based and highly accurate risk estimation. This study aimed to construct and verify a nomogram model to identify high-risk patients likely to develop S-AKI in elderly sepsis patients, based on variables available within 24 h of admission.

## Materials and methods

### Study design

This retrospective cohort study consisted of 849 old patients with sepsis between January 2015 and December 2021, and anonymized clinical data are collected from the Biobank of First Affiliated Hospital of Xi’an Jiaotong University (Xi’an, China).

### Patients

The inclusion criteria were as follows: (1) Elderly Patients(≥ 65 years old) were diagnosed as sepsis based on the sepsis 3.0 criteria, which defined sepsis as Sequential Organ Failure Assessment (SOFA) score ≥ 2 and documented or suspected infection[3]; (2) For patients admitted to hospital many times, only the first admission was considered for study. Exclusion criteria were as follows: (1) < 65 years old; (2) The length of stay in the hospital was less than 24 h; (3) A past medical history of chronic kidney disease (stage 4–5) or renal transplantation, or current hemodialysis, or acute kidney injury induced by other causes except sepsis; (4) Hematogical disorders.

### Data collection

The clinical variables in the study were collected from electronic medical records of the Biobank. (1) Demographic characteristics included age and gender. (2) The vital signs recorded in the first 24 h after admission included body temperature (T), heart rate (HR), respiratory rate (RR), and MAP. (3) Laboratory data were immediately available within 24 h after admission include WBC, neutrophil percentage (NEUT%), lymphocyte, monocyte, PLT, serum PCT, PTA, thrombin time (TT), international normalized ratio (INR), fibrinogen degradation products (FDP), D-Dimer (D-D), fibrinogen (FIB), activated partial thromboplastin time (APTT), prothrombin time (PT), AGR, globulin, albumin, bilirubin, urinary glucose, uric acid, Cystatin C, blood urea nitrogen (BUN), Cr, and total cholesterol (TC). (4) Therapies recorded in the first 24 h after admission included vancomycin, piperacillin-tazobactam, meropenem, and non-steroidal anti-inflammatory drugs (NSAIDs).

### Outcomes

The primary outcome was the events of AKI in sepsis patients. The diagnosis of AKI was based on the criteria of the Kidney Disease Improving Global Outcomes (KDIGO) classification(the serum creatinine level increases by ≥ 0.3 mg/dl (≥ 26.5 μmol/l) within 48 h, or the serum creatinine level increases ≥ 1.5 times over the baseline level within 7 days or cumulative 6 h urine output ≤ 0.5 ml/kg/h) [14]. Moreover, the secondary outcomes were the events of 30-day mortality and MAKE30 in sepsis patients. MAKE30 is a composition of death, new receipt of RRT, or PRD (defined as a final inpatient serum creatinine value greater than or equal to 200% of baseline) censored at hospital discharge or 30 days after inclusion, whichever occurred first. MAKE30 composite endpoints were defined as patients who met any of the three components of MAKE30. Baseline serum creatinine was defined as: (1) the first creatinine level obtained during the exposure period, (2) in the absence of the first creatinine level, the most recent creatinine measured within 12 months before hospitalization was obtained, (3) in the absence of measured values with these two approaches, an estimation is based on a previously described formula [creatinine = 0.74–0.2 (if female) + 0.003 × age (in years)] [15].

### Statistical analysis

The training group (75% of the sample size) was used to construct the prediction model, and the validation group (25% of the sample size) was used to verify the accuracy of the model. Continuous variables were expressed as median with quartile, while categorical variables are shown as percentage. Continuous variables were compared using Student’s *t* tests or the Mann–Whitney *U* tests as appropriate, and Pearson’s Chi-squared or Fisher’s exact tests were used to compare categorical variables. Univariate and multivariate logistic regression models were used to identify the independent predictors of S-AKI in the training cohort. The corresponding nomogram was constructed based on the results of multivariate logistic regression analysis, and then we constructed an online dynamic nomogram using the “DynNom” package. The AUC of the ROC curve was calculated to assess the discrimination of the nomogram in the training group and the validation group. To test whether the AUC is significant between the two groups, we used a two-sided test for ROC curves available online (http://vassarstats.net/roc_comp.html) [16]. The calibration curves were drawn to evaluate the consistency of the observed results and predicted probability. Decision curve analysis (DCA) was performed to assess the clinical net benefit of the predictive model. Finally, to assess the discrimination of the nomogram for 30-day mortality and MAKE30 in elderly patients, we performed the analysis of the secondary outcomes using ROC curves.

All analyses were performed using SPSS 26.0 and R version 4.1.2. *P* < 0.05 was considered statistically significant.

## Results

### Baseline characteristics of patients

**Fig. 1:**
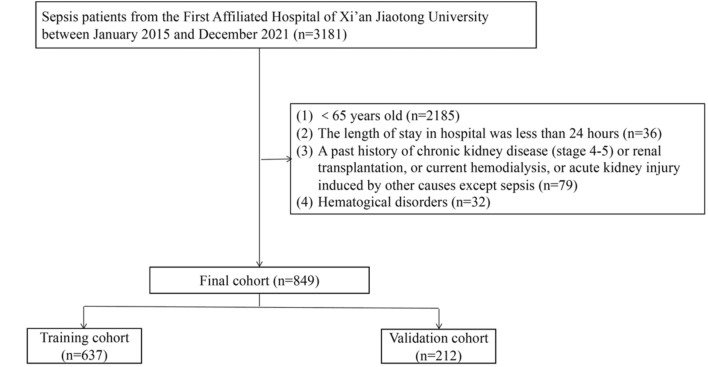
The flowchart of patient selection

As shown in Fig. 1, 849 old patients with sepsis were included, of whom 485 (57.1%) had AKI in the study. Moreover, the training set (75%, *n* = 637) and validation set (25%, *n* = 212) were selected randomly from the total cases. Similar demographic and clinical data between the training and validation groups are shown in Table 1, including demographic characteristics, vital signs, and laboratory examinations. The incidence of S-AKI in elderly patients with sepsis was 57.3% and 56.3% in the training and validation sets, respectively. The median age was 73 years (range, 68–79) and 72 years (range, 67–78), as well as 38.5–44.3% of patients were female in the training and validation sets, respectively.

**Table 1:** Baseline characteristics of the elderly patients with sepsis

Variable	Total (*n* = 849)	Training (*n* = 637)	Validation (*n* = 212)	*P* value
S-AKI, *n* (%)				0.92
No	364 (42.9)	272 (42.7)	92 (43.4)	
Yes	485 (57.1)	365 (57.3)	120 (56.3)	
Age	73 (68,79)	73 (68,79)	72 (67,78)	0.07
Gender, *n* (%)				0.15
Female	339 (39.9)	245 (38.5)	94 (44.3)	
Male	510 (60.1)	392 (61.5)	118 (55.7)	
Vital signs				
T(◦C)	36.6 (36.2,37.0)	36.6 (36.2,37.1)	36.6 (36.3,37.0)	0.89
RR (bpm)	20 (19,23)	20 (19,23)	20 (19,22)	0.11
HR (bpm)	90 (78,106)	92 (79,107)	87 (78,103)	0.06
MAP (mmHg)	91 (74,104)	92 (75,105)	90 (71,104)	0.21
Laboratory test				
WBC (× 10^9^/L)	8.95 (5.95,14.48)	9.14 (6.09,14.32)	8.24 (5.68,14.77)	0.54
Monocyte (× 10^9^/L)	0.40 (0.24,0.60)	0.40 (0.24,0.61)	0.40 (0.25,0.59)	0.76
NEUT (%)	86.8 (76.6,91.9)	86.9 (77.6,91.65)	86.4 (74.0,92.1)	0.52
Lymphocyte (× 10^9^/L)	0.76 (0.45,1.12)	0.76 (0.45,1.08)	0.76 (0.47,1.19)	0.46
PLT (× 10^9^/L)	141 (78,207)	142 (80,209)	135 (73,199)	0.36
PCT (ng/ml)	3.30 (0.56,17.05)	2.91 (0.52,14.02)	4.23 (0.65,30.15)	0.06
PTA (%)	71.0 (55.0,84.0)	70.0 (54.0,83.0)	73.0 (57.1,86.2)	0.10
TT (S)	14.10 (0.99,16.60)	14.40 (1.00,16.80)	1.24 (0.98,16.40)	0.18
INR	1.23 (1.11,1.42)	1.23 (1.12,1.42)	1.22 (1.11,1.39)	0.45
FDP (mg/L)	11.10 (5.29,24.85)	11.16 (5.34,26.00)	10.77 (5.09,20.19)	0.23
D-D (mg/L)	3.90 (1.91,8.13)	4.04 (1.91,8.95)	3.88 (1.93,6.87)	0.32
FIB (g/L)	4.24 (2.97,5.58)	4.15 (2.92,5.57)	4.54 (3.19,5.61)	0.21
APTT (S)	39.70 (34.60,45.70)	39.80 (34.70,45.90)	39.45 (34.41,45.10)	0.30
PT (S)	15.00 (13.30,16.80)	15.00 (13.30,16.90)	14.95 (13.30,16.48)	0.48
AGR	1.10 (1.00,1.40)	1.10 (1.00–1.40)	1.20 (1.00,1.50)	0.32
Globulin (g/L)	25.20 (21.60,29.20)	25.20 (21.90,29.15)	24.70 (21.12,29.35)	0.48
Albumin (g/L)	30.70 (25.60,39.00)	30.60 (25.45,39.50)	31.05 (25.90,38.00)	0.60
Bilirubin (mg/dl)	18.60 (11.50,36.10)	18.70 (11.80,36.85)	17.90 (10.83,34.53)	0.22
Urinary glucose (mmol/L)	7.03 (5.27,9.93)	7.06 (5.34,9.83)	6.92 (5.07,10.03)	0.66
UA (umol/L)	272.0 (188.5,376.5)	273.0 (188.0,382.5)	270.0 (192.5,361.8)	0.94
Cystatin C (mg/L)	1.29 (0.98,1.88)	1.29 (0.98,1.89)	1.28 (0.98,1.79)	0.94
BUN (mmol/L)	8.44 (5.34,14.33)	8.68 (5.43,14.00)	7.83 (5.16,15.28)	0.91
Cr (umol/L)	74.0 (53.0,127.5)	76.0 (54.0,130.0)	71.0 (51.0,118.8)	0.33
TC (mmol/L)	2.67 (1.96,3.52)	2.65 (1.92,3.51)	2.75 (2.03,3.59)	0.23

*S-AKI* sepsis-induced acute kidney injury, *T* body temperature, *HR* heart rate, *RR* respiratory rate, *MAP* mean arterial pressure, *WBC* white blood cell, *NEUT%* neutrophil percentage, *PLT* platelet, *PCT* serum procalcitonin, *PTA* prothrombin time activity, *TT* thrombin time, *INR* international normalized ratio, *FDP* fibrinogen degradation products, *D-D* D-Dimer, *FIB* fibrinogen, *APTT* activated partial thromboplastin time, *PT* prothrombin time, *AGR* albumin globulin ratio, *BUN* blood urea nitrogen, *Cr* creatinine, *TC* total cholesterol

Of 849 critically ill elderly people, 484 (57.0%) received either vancomycin (*n* = 146), piperacillin-tazobactam (*n* = 123), or meropenem (*n* = 215), and 141 (16.6%) received non-steroidal anti-inflammatory drugs (NSAIDs). As shown in Table 2, the risk of S-AKI was greatest for vancomycin (*P* < 0.001) compared with piperacillin-tazobactam (*P* = 0.012), but not significant for patients receiving meropenem (*P* = 0.353). Moreover, after exposure to NSAIDs, there was a greater risk of S-AKI in elderly sepsis patients (*P* < 0.001).

**Table 2:** Therapies of the elderly patients with sepsis during the first 24 h

Variable	Total (*n* = 849)	S-AKI (*n* = 485)	Non-AKI (*n* = 364)	*P* value
Vancomycin, *n* (%)	146 (17.2)	98 (20.2)	48 (13.1)	< 0.001
Piperacillin-tazobactam, *n* (%)	123 (14.5)	83 (17.1)	40 (11.0)	0.012
Meropenem, *n* (%)	215 (25.3)	117 (24.1)	98 (26.9)	0.353
NSAIDs, *n* (%)	141 (16.6)	95 (19.6)	46 (12.6)	< 0.001

*NSAIDs* non-steroidal anti-inflammatory drugs

### Univariate analysis results

All the demographic and clinical data were available within 24 h of admission. Univariate analyses in Table 3 revealed that age (1.025, 1.002–1.049), WBC (1.037, 1.014–1.060), NEUT (1.035, 1.021–1.049), PCT (1.030, 1.021–1.040), INR (10.316, 5.027–21.169), FDP (1.012, 1.006–1.019), D-D (1.039, 1.018–1.060), APTT (1.039, 1.022–1.057), PT (1.045, 1.019–1.071), UA (1.004, 1.002–1.005), Cystatin C (2.787, 2.139–3.631), BUN (1.129, 1.095–1.165), and Cr (1.018, 1.013–1.022) significantly increased in the S-AKI group than in the non-AKI group. While MAP (0.976, 0.968–0.985), Lymphocyte (0.613, 0.461–0.813), PLT (0.992, 0.990–0.994), PTA (0.968, 0.960–0.976), AGR (0.301, 0.189–0.477), and ALB (0.981, 0.965–0.997) were significantly lower in the S-AKI group.

**Table 3:** Univariate analysis of predictive variables of S-AKI in the training cohort

Variables	OR	95% CI	*P* value
Age (years)	1.025	1.002–1.049	0.037
Gender, *n* (%)	1.157	0.838–1.597	0.375
Vital signs			
T(◦C)	1.140	0.938–1.387	0.189
RR (bpm)	1.017	0.982–1.054	0.350
HR (bpm)	1.002	0.994–1.010	0.636
MAP (mmHg)	0.976	0.968–0.985	< 0.001
Laboratory test			
WBC (× 10^9^/L)	1.037	1.014–1.060	0.001
Monocyte (× 10^9^/L)	1.108	0.793–1.550	0.547
NEUT (%)	1.035	1.021–1.049	< 0.001
Lymphocyte (× 10^9^/L)	0.613	0.461–0.813	0.001
PLT (× 10^9^/L)	0.992	0.990–0.994	< 0.001
PCT (ng/ml)	1.030	1.021–1.040	< 0.001
PTA (%)	0.968	0.960–0.976	< 0.001
TT (S)	1.008	0.999–1.018	0.095
INR	10.316	5.027–21.169	< 0.001
FDP (mg/L)	1.012	1.006–1.019	< 0.001
D-D (mg/L)	1.039	1.018–1.060	0.038
FIB (g/L)	0.984	0.906–1.070	0.707
APTT (S)	1.039	1.022–1.057	< 0.001
PT (S)	1.045	1.019–1.071	0.001
AGR	0.301	0.189–0.477	< 0.001
Globulin (g/L)	1.007	0.982–1.032	0.604
ALB (g/L)	0.981	0.965–0.997	0.019
Bilirubin (mg/dl)	1.001	0.999–1.004	0.232
Urinary glucose (mmol/L)	0.994	0.981–1.006	0.314
UA (umol/L)	1.004	1.002–1.005	< 0.001
Cystatin C (mg/L)	2.787	2.139–3.631	< 0.001
BUN (mmol/L)	1.129	1.095–1.165	< 0.001
Cr (umol/L)	1.018	1.013–1.022	< 0.001
TC (mmol/L)	0.565	0.863–1.079	0.529

*T* body temperature, *HR* heart rate, *RR* respiratory rate, *MAP* mean arterial pressure, *WBC* white blood cell, *NEUT% *neutrophil percentage, *PLT* platelet, *PCT* serum procalcitonin, *PTA* prothrombin time activity, *TT* thrombin time, *INR* international normalized ratio, *FDP* fibrinogen degradation products, *D-D* D-Dimer, *FIB* fibrinogen, *APTT* activated partial thromboplastin time, *PT* prothrombin time, *AGR* albumin globulin ratio, *BUN* blood urea nitrogen, *Cr* creatinine, *TC* total cholesterol

### Predictors of S-AKI and nomogram development

**Fig. 2:**
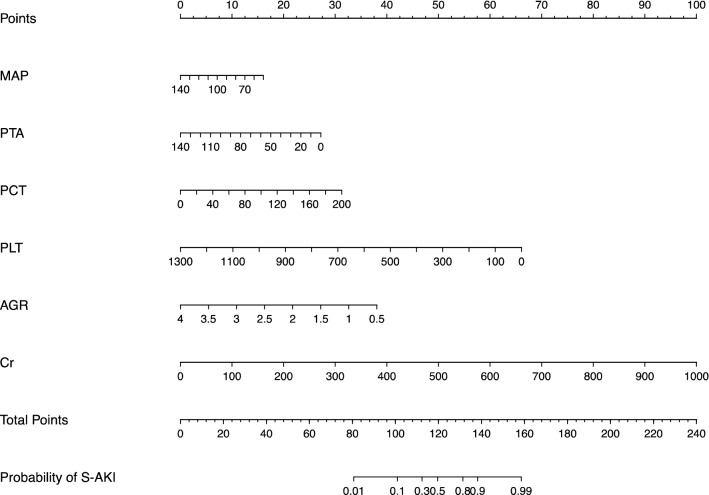
Nomogram to estimate the risk of S-AKI in elderly patients with sepsis. *S-AKI* Sepsis-induced acute kidney injury, *MAP* mean arterial pressure, *AGR* albumin globulin ratio, *PTA* prothrombin time activity, *PCT* serum procalcitonin, *PLT* platelet, *Cr* creatinine

Multivariate logistic regression analyses were performed for the significant variables from the univariate analyses, including age, gender, MAP, WBC, NEUT, lymphocyte, PLT, PCT, PTA, INR, FDP, D-D, APTT, PT, Cystatin C, BUN, UA, Cr, AGR, and ALB. In multivariate analysis, MAP (0.979, 0.969–0.989), AGR (0.277, 0.161–0.477), PTA (0.977, 0.968–0.986), PCT (1.019, 1.009–1.028), Cr (1.012, 1.008–1.016), and PLT (0.994, 0.992–0.996) were identified as the independent predictors for S-AKI in old patients suffering from sepsis (*P* < 0.001, Table 4). As shown in Fig. 2, a nomogram based on these characteristics was developed to predict S-AKI in elderly patients with sepsis. Furthermore, we used the “DynNom” package to provide an online version of this nomogram, facilitating its widespread use by physicians and researchers (https://sepsis-acute-kidney-injury.shinyapps.io/S-AKI/).

**Table 4:** Multivariate logistic regression analysis of independent predictors of S-AKI in the training cohort

Variables	OR	95% CI	*P* value
MAP (mmHg)	0.979	0.969–0.989	< 0.001
AGR	0.277	0.161–0.477	< 0.001
PTA (%)	0.977	0.968–0.986	< 0.001
PCT (ng/ml)	1.019	1.009–1.028	< 0.001
PLT (× 10^9^/L)	0.994	0.992–0.996	< 0.001
Cr (umol/L)	1.012	1.008–1.016	< 0.001

*MAP* mean arterial pressure, *AGR* albumin globulin ratio, *PTA* prothrombin time activity, *PCT* serum procalcitonin, *PLT* platelet, *Cr* creatinine

### Verification of the prediction model

**Fig. 3:**
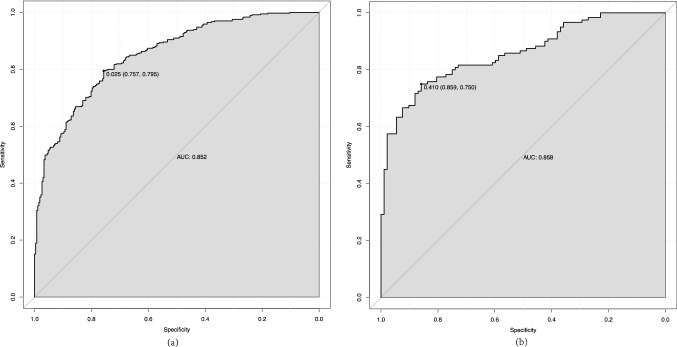
The ROC curve of the nomogram for predicting S-AKI in elderly sepsis patients. **a** ROC curves in training set; **b** ROC curves in validation set

**Fig. 4:**
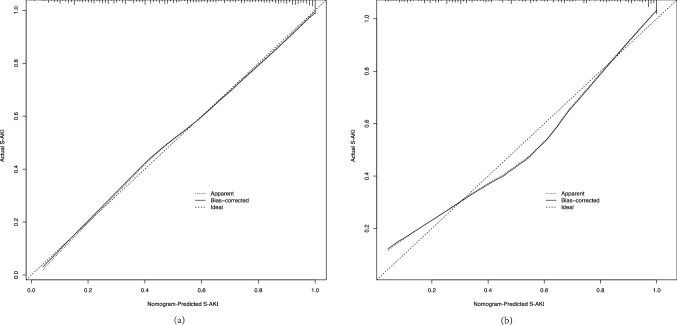
Calibration curves of the predicted nomogram. Evaluation of the predictive performance for estimating the risk of S-AKI of the nomogram in the training set (**a**) and validation set (**b**)

**Fig. 5:**
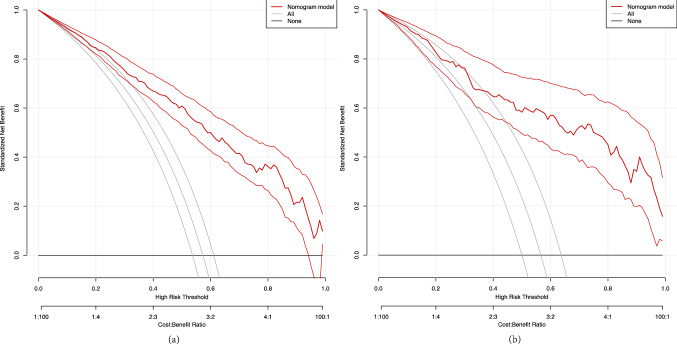
Decision curve analysis of the nomogram. **a** Decision curve analysis in the training set; **b** Decision curve analysis in validation set

As shown in Fig. 3, ROC curve analyses revealed that the model had a good ability to predict S-AKI from elderly patients with sepsis in the training (AUC = 0.852) and validation (AUC = 0.858) cohorts. Moreover, the two cohorts were non-significant (*Z* = − 0.205, *P* = 0.84) using a two-sided test for ROC curves available online (http://vassarstats.net/roc_comp.html). The calibration curve of the model for the training and validation cohorts showed that the predictive probabilities were in consistent agreement with the observation results, indicating a successful calibration (Fig. 4). As shown in Fig. 5, the DCA demonstrated that the nomogram had superior overall net benefit within the wide and practical ranges of threshold probabilities, indicating high potential for clinical utility.

### The secondary outcome analysis using the prediction model

**Fig. 6:**
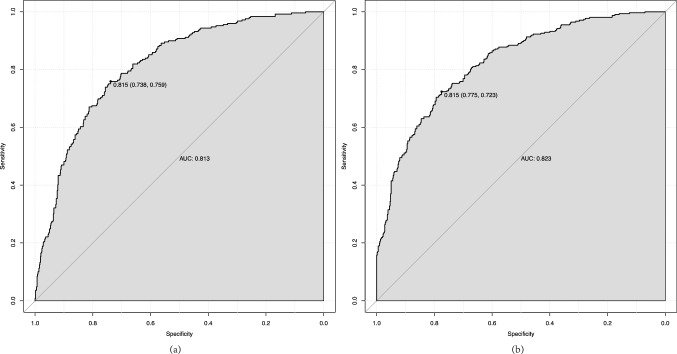
Receiver operating characteristic curve of the nomogram model for 30-day mortality **a** and MAKE30 **b**. MAKE30, major adverse kidney events within 30 days

Overall, the MAKE30 composite endpoints were reached in 311 of the 849 (36.6%) patients. For the individual components, the incidence was 249 (29.3%) for 30-day mortality, 103 (12.1%) for new RRT, and 43 (5.1%) for persistent renal dysfunction, respectively. As shown in Fig. 6, ROC curve analyses revealed that the prediction model had a good ability to predict the occurrence of 30-day mortality (AUC = 0.813) and MAKE30 (AUC = 0.823) in elderly patients with sepsis.

## Discussion

In the present study, we constructed a simple nomogram model based on those independent predictors (MAP, PCT, PLT, PTA, AGR, and Cr) within 24 h of admission, which performed well for predicting S-AKI in elderly patients with sepsis. In addition, the use of prognostic models will allow for timely, high-quality, person-centered supportive care and a care bundle for S-AKI. Therefore, the model might improve the outcome and reduce mortality in elderly patients with sepsis.

A retrospective study including 146,148 patients across nine regional central hospitals in China reported AKI in 47.1% of sepsis cases [17]. In this study, we discovered a higher incidence of S-AKI in elderly sepsis patients, as high as 57.1%, because of their older age. In addition, this study revealed that vancomycin, piperacillin-tazobactam, or NSAIDs added to the risk of S-AKI but not meropenem in elderly sepsis patients. A prospective multicenter Sapphire study (NCT01209169; ClinicalTrials.gov) indicated that the combination of piperacillin–tazobactam and vancomycin is associated with possible increased S-AKI and long-term adverse outcomes [18]. NSAIDs can result in AKI due to reducing renal blood flow, causing tubular obstruction through crystal deposition, and inducing direct cytotoxicity and cell-mediated immune injury mechanisms [19]. Furthermore, Older age and chronic kidney disease are also related to the onset of AKI during NSAID use [19]. Physiologically, multiple factors may contribute to diminished renal function with the increase of age, including the atrophy and degeneration of renal tubular epithelial cells, thickened basement membrane, and decreased renal blood flow.

Sepsis is the most common cause of AKI in elderly patients [20]. Increasing studies discovered that ischemia–reperfusion injury is not the only mechanism of S-AKI, but rather multiple mechanisms contribute to the pathogenesis of AKI, including inflammation, microcirculatory dysfunction, and metabolic reprogramming [21]. During sepsis, inflammatory mediators are released in the intravascular compartment and damage the vascular endothelium. Then, activated platelet aggregation hyperactivates the coagulation system, which plays a significant role in the microcirculatory alterations contributing to kidney injury [22]. It is widely known that during sepsis, suppression of aerobic glycolysis and augmentation of oxidative phosphorylation (OXPHOS) result in a lower vulnerability to AKI [23]. However, inflammatory mediators induced significant mitochondrial injury, accelerating the metabolic reprogramming switch from OXPHOS to aerobic glycolysis and thereby mediating kidney damage [21].In addition, a dysfunctional immune system, altered cytokine production, and coagulation abnormalities in old age increased the incidence of S-AKI in sepsis [4].

In the study, logistic regression analysis showed that MAP, PCT, PLT, PTA, AGR, and Cr were independent predictors for S-AKI in elderly sepsis patients. Those biomarkers just reflected that S-AKI resulted from a cross-talk between ischemia–reperfusion injury, inflammatory cascade activation, deranged coagulation pathways, and malnutrition [21, 24, 25]. Our study indicated that low MAP was an independent predictor for S-AKI, indicating renal tissue ischemia and hypoxia. According to classical theories, renal hypotension and associated ischemia, as well as a lack of oxygen, are the main pathological mechanisms underlying AKI [6]. Additionally, PCT has been widely used to diagnose bacterial infections and guide antibiotic therapy in sepsis patients [26, 27]. This study had similar findings to previous studies that PCT was more accurate at predicting S-AKI than other inflammatory biomarkers, such as WBC, lymphocytes, monocytes, and NEUT [27, 28]. PCT can cause mesangial cell apoptosis and direct cytotoxicity by boosting the synthesis of proinflammatory cytokines [29]. Moreover, PCT acted as a monocyte chemoattractant at the site of inflammation, contributing to inflammation-mediated cell damage [30]. Thus, a high level of PCT was expected to represent the occurrence of S-AKI in sepsis patients [10].

Inflammatory activities can damage the vascular endothelium, activating platelets first and causing coagulation disorders subsequently [24, 31]. Nevertheless, platelets not only regulate hemostasis but also interact with other immune cells to affect the immunological and inflammatory response[32, 33]. In the pathogenesis of S-AKI, platelets aid in stimulating endothelial cells as well as attracting and activating leukocytes during the inflammatory response [31]. Previous studies have discovered that platelets play a critical role in S-AKI by inducing renal cell apoptosis in the septic mouse [34, 35]. Early evidence from animal and human research, as well as randomized clinical trials, suggested that antiplatelet treatments may reduce the risk of AKI [31]. Furthermore, excessive platelets and coagulation factors consumption aggravated hyperfibrinolysis, resulting in coagulation disorders. However, few studies utilized coagulative biomarkers to predict S-AKI development in elderly patients with sepsis. A prolonged APTT can be confused by a variety of factors in patients in the hospital, including vitamin K deficiency, transient lupus inhibitors, high factor VIII levels, and heparin contamination [36]. In this study, we observed that PTA was identified as an independent predictor for S-AKI. A wide range of thromboplastin reagents may cause large differences between laboratories in PT results. PTA or INR could therefore serve as a supplement to PT reporting and better predict the risks associated with S-AKI [37, 38]. Additionally, platelet count and PTA were highly predictive of 28-day mortality in patients with sepsis and coagulopathy complications [39]. Furthermore, thrombocytopenia and decreased PTA were associated with organ dysfunction and were related to poor clinical outcomes in sepsis patients [39, 40].

The interactions between inflammation response and malnutrition are highly associated with the pathogenesis of S-AKI in elderly sepsis patients [10]. First, AGR, a predictor of cancer progress and cancer-related mortality in previous studies, mainly reflected the state of nutrition, inflammation, and immunity [41]. After the creation of a multivariate model, this study first indicated that AGR was also an independent predictor of S-AKI. Serum albumin is associated with malnutritional status, as well as being diluted and inhibited during inflammation [42]. During the hypermetabolic and hypercatabolic state in sepsis, albumin catabolism was enhanced. Thus, hypoalbuminemia is a typical clinical symptom in sepsis patients and contributes to the mortality risk among such patients [10, 42, 43]. Serum albumin might play a significant role in reducing the incidence of kidney injury via anti-inflammation, anti-oxidation, and maintenance of renal vascular endothelium function integrity [42, 43]. Serum globulin is significant in evaluating the overall state of immunity and inflammation. In the stage of sepsis, albumin can indirectly contribute to immunity by providing nitrogen for globulin production, resulting in hypoalbuminemia and elevated globulin concentration [44]. Pai et al. indicated that higher serum globulin (≥ 38 g/L) contributed to a higher infection-related mortality risk in 104,154 incident hemodialysis patients [45]. Thus, a lower AGR can result from a combination of low albumin and high globulin, and the combined biomarker might be a robust predictor of S-AKI in elderly sepsis patients. Second, as a functional marker of AKI, Cr is a small molecule generated in muscle. Up until now, AKI has been defined by a rapid increase in serum Cr, a decrease in urine output, or both, indicating that Cr has a significantly predictive value for AKI [46]. Furthermore, Hidayati et al. found that cystatin C was not superior to serum Cr in detecting neonatal AKI [47].

In this study, we constructed and validated a simple nomogram model, based on variables available within 24 h of admission, to predict S-AKI in elderly patients with sepsis. The designed nomogram performed well in the discrimination, calibration, and clinical application after verification. In addition, this nomogram provided valuable information for determining the appropriate therapy options for high-risk patients likely to develop S-AKI. Here we cite an example to show how to use the nomogram model, assuming an elderly sepsis patient (≥ 65 years) with a MAP of 70 mmHg, a PCT of 40 ng/ml, a PTA of 90%, an AGR of 1, a PLT of 200 × 10^9^/L, and a Cr of 300 umol/L. According to Fig. 2, the score corresponding to each parameter on the “Points” axis is obtained. The overall score is calculated as the sum of points for all parameters [13 (MAP) + 7 (PCT) + 10 (PTA) + 32 (AGR) + 54 (PLT) + 30(Cr) = 146]. This score corresponds to an approximately 96% risk of developing S-AKI. In another way, you can use the online version to attain the same result easily (https://sepsis-acute-kidney-injury.shinyapps.io/S-AKI/).

In this study, 311 (36.6%) elderly patients experienced the MAKE30 composite outcome, making the incidence of MAKE30 higher than that in adult patients with sepsis (28.8%) [15]. Moreover, 249 (29.3%) elderly patients experienced 30-day mortality, which was higher than adult patients with sepsis (22.7%) or patients aged 60–64 (26%) [4, 15]. Finally, we also discovered that the nomogram model had perfect predictive power for predicting 30-day mortality (AUC = 0.813) and MAKE30 (AUC = 0.823) in elderly sepsis patients.

### Limitations

Furthermore, this study had several limitations: (1) this was a single-center study with small sample size, so there was a possibility that selection bias influenced the results. (2) This was a retrospective study for seven years. Therefore, we used uniform diagnostic criteria for the differential diagnosis of sepsis, AKI, and MAKE30 to ensure the omogeneity of the data. However, during this time, the decision-making procedure for sepsis management evolved substantially, which is a perplexing element affecting S-AKI development. In early empiric antimicrobial therapy during sepsis, a great deal of progress has been made in the fields of pharmacokinetics and pharmacodynamics, drug dosing, therapeutic drug monitoring, and antimicrobial resistance [48]. (3) The nomogram was developed and validated by the same database. Furthermore, the model was constructed from a training group with a much smaller testing population. Therefore, it is necessary to assess its potential by testing the model in different and larger populations. Moreover, further multi-center prospective studies would be needed to verify the results.

## Conclusions

In addition, the designed nomogram, based on MAP, AGR, PTA, PCT, Cr, and PLT, performed well in the discrimination, calibration, and clinical application. This model is of great importance for clinicians to make personalized management for S-AKI in elderly sepsis patients.

## Supplementary Information

Below is the link to the electronic supplementary material.

Supplementary file1 (XLSX 186 KB)

Supplementary file2 (XLSX 123 KB)

Supplementary file3 (XLSX 47 KB)

## Data Availability

The original contributions presented in the study are included in the article/Supplementary Material, further inquiries can be directed to the corresponding author/s.

## References

[1] Foley C, Bloomer M, Hutchinson AM (2022). Factors that influence intensive care admission decisions for older people: a systematic review. Aust Crit Care.

[2] Nielsson MS, Christiansen CF, Johansen MB (2014). Mortality in elderly ICU patients: a cohort study. Acta Anaesthesiol Scand.

[3] Singer M, Deutschman CS, Seymour CW (2016). The third international consensus definitions for sepsis and septic shock (sepsis-3). JAMA.

[4] Starr ME, Saito H (2014). Sepsis in old age: review of human and animal studies. Aging Dis.

[5] Xie Y, Tian R, Jin W (2020). Antithrombin III expression predicts acute kidney injury in elderly patients with sepsis. Exp Ther Med.

[6] Poston JT, Koyner JL (2019). Sepsis associated acute kidney injury. BMJ.

[7] Xie Y, Huang P, Zhang J (2021). Biomarkers for the diagnosis of sepsis-associated acute kidney injury: systematic review and meta-analysis. Ann Palliat Med.

[8] Suzuki C, Tanida I, Oliva Trejo JA (2019). Autophagy deficiency in renal proximal tubular cells leads to an increase in cellular injury and apoptosis under normal fed conditions. Int J Mol Sci.

[9] Wang JJ, Chi NH, Huang TM (2018). Urinary biomarkers predict advanced acute kidney injury after cardiovascular surgery. Crit Car.

[10] Chen L, Wu X, Qin H (2021). The PCT to albumin ratio predicts mortality in patients with acute kidney injury caused by abdominal infection-evoked sepsis. Front Nutr.

[11] Yue S, Li S, Huang X (2022). Construction and validation of a risk prediction model for acute kidney injury in patients suffering from septic shock. Dis Markers.

[12] Xu Z, Cheng B, Fu S (2019). Coagulative biomarkers on admission to the ICU predict acute kidney injury and mortality in patients with septic shock caused by intra-abdominal infection. Infect Drug Resist.

[13] Zhang X, Ye B, Mao W (2022). Major adverse kidney events within 30 days in patients with acute pancreatitis: a tertiary-center cohort study. HPB (Oxford).

[14] Stevens PE, Levin A (2013). Evaluation and management of chronic kidney disease: synopsis of the kidney disease: improving global outcomes 2012 clinical practice guideline. Ann Intern Med.

[15] Mele A, Cerminara E, Habel H (2022). Fluid accumulation and major adverse kidney events in sepsis: a multicenter observational study. Ann Intensive Care.

[16] Hanley JA, McNeil BJ (1982). The meaning and use of the area under a receiver operating characteristic (ROC) curve. Radiology.

[17] Xu X, Nie S, Liu Z (2015). Epidemiology and clinical correlates of AKI in Chinese hospitalized adults. Clin J Am Soc Nephrol.

[18] Kane-Gill SL, Ostermann M, Shi J (2019). Evaluating renal stress using pharmacokinetic urinary biomarker data in critically ill patients receiving vancomycin and/or piperacillin-tazobactam: a secondary analysis of the multicenter sapphire study. Drug Saf.

[19] Zhang X, Donnan PT, Bell S (2017). Non-steroidal anti-inflammatory drug induced acute kidney injury in the community dwelling general population and people with chronic kidney disease: systematic review and meta-analysis. BMC Nephrol.

[20] Hoste EA, Bagshaw SM, Bellomo R (2015). Epidemiology of acute kidney injury in critically ill patients: the multinational AKI-EPI study. Intensive Care Med.

[21] Peerapornratana S, Manrique-Caballero CL, Gomez H (2019). Acute kidney injury from sepsis: current concepts, epidemiology, pathophysiology, prevention and treatment. Kidney Int.

[22] Engelmann B, Massberg S (2013). Thrombosis as an intravascular effector of innate immunity. Nat Rev Immunol.

[23] Ji R, Chen W, Wang Y (2021). The warburg effect promotes mitochondrial injury regulated by uncoupling protein-2 in septic acute kidney injury. Shock.

[24] Fani F, Regolisti G, Delsante M (2018). (2018) Recent advances in the pathogenetic mechanisms of sepsis-associated acute kidney injury. J Nephrol.

[25] Wiedermann CJ, Wiedermann W, Joannidis M (2010). Hypoalbuminemia and acute kidney injury: a meta-analysis of observational clinical studies. Intensive Care Med.

[26] Wirz Y, Meier MA, Bouadma L (2018). Effect of procalcitonin-guided antibiotic treatment on clinical outcomes in intensive care unit patients with infection and sepsis patients: a patient-level meta-analysis of randomized trials. Crit Care.

[27] Fu G, Zhan HC, Li HL (2021). Association between procalcitonin and acute kidney injury in patients with bacterial septic shock. Blood Purif.

[28] Kan WC, Huang YT, Wu VC (2021). Predictive ability of procalcitonin for acute kidney injury: a narrative review focusing on the interference of infection. Int J Mol Sci.

[29] Jeeha R, Skinner DL, De Vasconcellos K (2018). Serum procalcitonin levels predict acute kidney injury in critically ill patients. Nephrology (Carlton).

[30] Wiedermann FJ, Kaneider N, Egger P (2002). (2002) Migration of human monocytes in response to procalcitonin. Crit Care Med.

[31] Jansen MPB, Florquin S, Roelofs J (2018). The role of platelets in acute kidney injury. Nat Rev Nephrol.

[32] Nicolai L, Massberg S (2020). Platelets as key players in inflammation and infection. Curr Opin Hematol.

[33] Joffre J, Hellman J, Ince C (2020). Endothelial responses in sepsis. Am J Respir Crit Care Med.

[34] Li X, Li Y, Shen K (2019). The protective effect of ticagrelor on renal function in a mouse model of sepsis-induced acute kidney injury. Platelets.

[35] Lv D, Zhang Y, Wang C (2022). platelets derived transthyretin participate in the development of sepsis associated acute kidney injury by inducing oxidative stress and apoptosis of renal tubular epithelial cells. Shock.

[36] Yuan S, Ferrell C, Chandler WL (2007). Comparing the prothrombin time INR versus the APTT to evaluate the coagulopathy of acute trauma. Thromb Res.

[37] Li S, Liu Z, Wu H (2019). The product value of serum albumin and prothrombin time activity could be a useful biomarker for severity prediction in AP: an ordinal retrospective study. Pancreatology.

[38] Luo HC, You CY, Lu SW (2021). Characteristics of coagulation alteration in patients with COVID-19. Ann Hematol.

[39] Iba T, Nisio MD, Levy JH (2017). New criteria for sepsis-induced coagulopathy (SIC) following the revised sepsis definition: a retrospective analysis of a nationwide survey. BMJ Open.

[40] Thiery-Antier N, Binquet C, Vinault S (2016). Is thrombocytopenia an early prognostic marker in septic shock?. Crit Care Med.

[41] Lv GY, An L, Sun XD (2018). Pretreatment albumin to globulin ratio can serve as a prognostic marker in human cancers: a meta-analysis. Clin Chim Acta.

[42] Takegawa R, Kabata D, Shimizu K (2019). Serum albumin as a risk factor for death in patients with prolonged sepsis: an observational study. J Crit Care.

[43] Sheng S, Zhang YH, Ma HK (2022). Albumin levels predict mortality in sepsis patients with acute kidney injury undergoing continuous renal replacement therapy: a secondary analysis based on a retrospective cohort study. BMC Nephrol.

[44] Lu J, Xun Y, Yu X (2020). Albumin-globulin ratio: a novel predictor of sepsis after flexible ureteroscopy in patients with solitary proximal ureteral stones. Transl Androl Urol.

[45] Pai AY, Sy J, Kim J et al (2021) Association of serum globulin with all-cause mortality in incident hemodialysis patients. Nephrol Dial Transplant 10.1093/ndt/gfab292 PMC9494081 34617572

[46] Ronco C, Bellomo R, Kellum JA (2019). Acute kidney injury. Lancet.

[47] Hidayati EL, Utami MD, Rohsiswatmo R (2021). Cystatin C compared to serum creatinine as a marker of acute kidney injury in critically ill neonates. Pediatr Nephrol.

[48] Póvoa P, Moniz P, Pereira JG (2021). Optimizing antimicrobial drug dosing in critically Ill patients. Microorganisms.

